# Exploring experiences among adopters during the diffusion of a novel dance intervention in Sweden

**DOI:** 10.1080/17482631.2018.1438697

**Published:** 2018-02-21

**Authors:** Noomi Carlsson, Agneta Kullberg, Ida-Klara Johansson, Paula Bergman, Janna Skagerström, Agneta Andersson

**Affiliations:** ^a^ Public Health & Healthcare, Regional Executive Office, Jönköping, Sweden; ^b^ Division of Community Medicine, Social Medicine and Public Health Science, Department of Medical and Health Sciences, Linköping University, Linköping, Sweden; ^c^ Research & Development Unit in Local Health Care, and Department of Medicine and Health Sciences, Linköping University, Linköping, Sweden

**Keywords:** Implementation, psychosomatic, diffusion, innovation, dance intervention

## Abstract

There is a demand for interventions aimed at adolescent girls with psychosomatic problems. In 2013, positive results were reported from a dance intervention programme addressing girls with internalizing problems. The research team behind the intervention immediately received requests from municipalities and county councils interested in using the intervention. From an implementation point of view it is unclear what made the intervention spread without an active plan. The aim of this study was to explore adopters’ experiences about the diffusion and initiation of a public health intervention targeting adolescent girls with internalizing problems. Interviews were conducted with 12 people who were engaged in initiating the intervention in different settings. Data were analysed using conventional content analysis, yielding three categories: perceived appeal and trustworthiness, convenient information, and contextual factors. The results reflected that the participants found that there was a need for an intervention and found the dance intervention to be evidence based and not too complex to perform. Further, there was available information on the project which could easily be distributed to decision makers and others. When initiating the intervention, factors related to economy, possibility for collaboration and recruitment were of importance.

## Introduction

Psychosomatic symptoms and mental health problems among adolescents have increased rapidly worldwide in recent years (Bor, Dean, Najman, & Hayatbakhsh, 2014). According to the World Health Organization (2012, 2016), Sweden has seen a particularly large increase in mental health problems. The prevalence of self-rated psychosomatic problems among Swedish adolescents has increased in the past few decades, as has the number of contacts with inpatient, outpatient and primary care due to psychosomatic illness (Folkhälsomyndigheten, 2016). All health care in Sweden is publicly funded by taxes and mainly organized by two authorities: county councils and local municipalities. Throughout Sweden, these authorities are trying to tackle this complex public health issue and the political pressure on decision makers is considerable.

A dance intervention programme for adolescent girls with internalizing problems, i.e., with stress and psychosomatic symptoms, was developed and a randomized controlled trial was performed by a Swedish research team and published in *JAMA Pediatrics* in 2013 (Duberg, Hagberg, Sunvisson, & Möller, 2013). Evaluations show that the intervention improved self-rated health at 12 and 20 month follow-up (mean difference 0.62 and 0.40) and was a positive experience (Duberg et al., 2013). In a qualitative study, girls who had participated in the dance intervention expressed that the intervention had given them a feeling of being free, and increased self-trust and confidence (Duberg, Möller, & Sunvisson, 2016). The dance intervention, organized as a complement to the school health services, was demonstrated to be cost-effective compared with usual school health services alone (Philipsson, Duberg, Möller, & Hagberg, 2013).

Despite the relatively limited scientific evidence at the time, the intervention received widespread media coverage and the research team immediately received requests to educate dance instructors and instruct them how to set up the intervention in other locations across Sweden. In conjunction with the research process, the research team at Örebro University offered courses for dance instructors according to the dance intervention programme. Although the intervention has spread to several municipalities, it is still unclear what characteristics of the intervention made it spread without an active dissemination plan.

The gap between research and practice has been widely reported and discussed. Traditionally, the public health impact of intervention studies has not been the concern of health researchers (Bauer, Damschroder, Hagedorn, Smith, & Kilbourne, 2015). However, interest in implementation science has grown in the past few decades to study what influences the implementation of new practices (including interventions, programmes and services) and what strategies might be effective to support implementation. Numerous so-called determinant frameworks have been developed to describe barriers and facilitators that influence the implementation and use of new practices (Nilsen, 2015). Most of these frameworks describe three types of determinants that influence implementation: perceived attributes of the practice (e.g., complexity and relative advantage in relation to the current practice); individual characteristics of the users of the new practice (e.g., attitudes, beliefs and motivation concerning the new practice); and contextual factors (e.g., organizational support and support from colleagues and time constraints) (Cochrane, Olson, Murray, Dupuis, Tooman, Hayes., 2007; Damschroder et al., 2009; Grol, Wensing, & Eccles, 2005; Rycroft-Malone & Bucknall., 2010). The frameworks also address various strategies that support implementation, ideally accounting for as many determinants as possible to achieve successful implementation of a new practice (Bauer et al., 2015).

Despite the fact that implementation science has identified many implementation barriers, new practices sometimes spread by themselves, without the use of any conscious strategies. Rogers’ diffusion of innovations theory (Rogers, 2003) deals with the spontaneous spread of new practices, i.e., innovations. The theory posits that diffusion of new practices in a social context, e.g., health care, is influenced by a number of factors, including the setting, the users, the perceived need for the intervention and external influences. Several implementation frameworks are influenced by the work of Rogers and have identified similar factors as being important (Damschroder et al., 2009; Greenhalgh, Robert, Bate, Macfarlane, & Kyriakidou, 2005; Gurses et al., 2010). However, more research is needed to understand how public health interventions are perceived and spread within politically controlled organizations outside the health-care sector.

The aim of the present study was to explore adopters’ experiences of the diffusion and initiation of a public health intervention targeting adolescent girls with internalizing problems, i.e., stress and psychosomatic symptoms.

## Methods

### Recruitment and informants

Individuals who participated in the courses to become a dance instructor during 2013–2014 were contacted. The dance instructors were asked to provide contact information for their stakeholders in decision making and/or collaboration partners in the dance intervention. Among 27 potential informants provided by the dance instructors, 18 were strategically selected and asked to participate in the study. Most of the selected informants had a directorship. The nine potential informants who were not asked to participate were on sick leave, had a new job or had a peripheral position in relation to the dance project. To generate a variety of experiences of the intervention process, selection was based on the organizational role of the respondents. Further, heterogeneity regarding the municipality or counties where the respondents worked was sought to capture the diffusion of the intervention in different settings. The potential informants were informed about the study design and purpose via e-mail in November 2014 and a reminder was sent in December 2014.

Twelve of the 18 potential informants agreed to be interviewed and were contacted by telephone to schedule a semi-structured telephone interview. The informants represented four different regions in southern Sweden and were all employed in municipalities or county councils. Most were employed either in public health departments or in children and education or culture departments. The informants’ professions included public health planner, physiotherapist, and manager or coordinator in school health, public health or health care. Eleven of the 12 respondents were women. Most of the informants were community officials, decision makers and/or promoters of the dance project. Three of them had also participated in the course to become a dance instructor. Five people did not answer our e-mail or declined to participate.

### Procedure

The interview guide was based on Rogers’ attributes of innovations, relative advantage, compatibility, complexity, trialability and observability (Rogers, 2003). For example, the question “How do you view the dance intervention in relation to how you have worked before?” was intended to capture relative advantage, “Did introduction of the dance intervention meet your expectations to intervene with the target group?” to capture compatibility and “Describe your experiences of initializing the dance intervention” to capture complexity.

The guide contained questions on initial contact with the dance intervention, reasons for considering the intervention, response to the intervention, initiation process and goals of the intervention. Twelve telephone interviews were conducted by N.C. and A.K., six by each author. The interviews were recorded and lasted on average 27 minutes (range 19–43 minutes). All interviews were performed in Swedish and transcribed by a professional transcriptionist, and selected quotations were then translated into English by a professional translator.

### Analysis

The data were analysed using conventional content analysis according to Hsieh and Shannon (2005). The interviews were transcribed verbatim and read by all authors several times to obtain a sense of the whole. Initially, the transcribed text was coded by N.C. and A.K. separately. The data material was structured into meaning-bearing units and then condensed, and subcategories and main categories were constructed. Throughout the analysis process, all the main categories and codes were discussed among all the authors until consensus was reached. Verbatim quotations are provided to illustrate each subcategory. Selected citations were translated by a professional translator.

### Ethics

The study was approved by the Regional Ethical Board in Uppsala, Sweden (Dnr 2014/090). The participants were informed that the material from the interviews would be presented in a way that guaranteed confidentiality. Informed consent was obtained by the participants’ positive replies to the e-mail. Verbal consent was also obtained at the start of the interview. Participants were also told that they could discontinue their participation at any time.

## Results

The analysis resulted in three main categories and 10 sub-categories (Table I). Verbatim quotations from the interview persons (IPs) are used to illustrate the findings and show a link to the original data, and are numbered at random.

**Table I.** Overview of the results presented as categories and sub-categories.

Main category	Sub-category
Perceived appeal and trustworthiness	Pent-up demand
	Perceived as an evidence-based method
	Endorsement of own beliefs
Convenient information	Information channels
	Transferable concept
Contextual factors	Organizational position
	Collaborative strategies
	Personal competence and qualities
	Time constraints
	Reach of the intervention

### Perceived appeal and trustworthiness

The dance intervention filled a clear and pent-up demand for a solution to a public health problem. In addition, the intervention was perceived as trustworthy by the fact that positive results were published in a scientific journal, and it also endorsed the informants’ own beliefs and expectations.

#### Pent-up demand

Enhanced pressure from the political leadership about handling the increasing prevalence of psychological health problems among adolescents was described by the participants. This was seen as a challenge within their professional assignment as they perceived a paucity of options. The informants expressed that, at the time when they first heard about the dance intervention, they had limited or no interventions to offer the target group.

Yes, well the issue was actually raised in political forums because mental ill health is a major problem with us, so it has actually been a prioritized area for a number of years. (IP1)

The respondents recognized the dance project as an intervention that, from several aspects, was realistic to achieve and thus offered a possible solution to face the challenge described above. Described advantages of the dance intervention were the focus on a distinct health-promoting activity and the possibilities to perform the intervention within a reasonable budget and training initiative.

#### Perceived as evidence based

Another benefit of the intervention expressed by the informants was that research had been conducted and published in a highly regarded scientific journal, showing positive results. The fact that research had been conducted gave the respondents a sense of trust in the method regarding its potential to have a positive impact on the target group. The opportunity to use and adopt a developed and assessed intervention gave a sense of quality and safety in the planning and implementation of the dance classes.

It is something that has been researched, there are good results, it is a target group that we are to prioritize. […] I think that it has massive importance that it has been researched. (IP11)

#### Endorsement of own beliefs

For some of the informants, the positive research results strengthened their own perception of dance as a positive influence on health. It was also something that they expressed as a strong argument with colleagues and political leaders.

Ah, personally I am really passionate about this and I have always believed in it, so it was fantastic that there is now scientific proof that dance is a good thing. (IP6)

### Convenient information

The informants picked up information about the innovation through several channels, through the Internet, from colleagues or through informal channels. How the innovation was presented played a vital role, because it was easy to repeat the message and pass it on to others.

#### Information channels

The media seems to have played a vital role in the diffusion of the intervention. Apart from the articles published in a scientific journal and presentations given at scientific meetings, the intervention has been positively depicted in local newspapers as well as on news reports on television, radio and Internet (YouTube). The informants described that their initial contact with the dance intervention was primarily through networks connected to their profession, newsletters and e-mail lists. It may also have been a direct call from a manager or political leader to assess the information about the dance intervention and give an opinion on whether it could be useful in their organization.

#### Transferable concept

A facilitating circumstance for the diffusion of the intervention was that the information about the dance intervention was perceived as appealing to the informants. The short film clip explaining the intervention made the information accessible and easy to pass on via e-mail or to show in a meeting.

The day before there had been a report on it on Aktuellt [the current affairs TV programme]. Because the research had been published and was internationally recognized, the link was active for a long time. So we could send it to other people to help describe what it was. (IP11)

### Contextual factors

Decision-making powers in implementing the dance project varied among the informants but they all faced the need to firmly establish the idea inside their organization as well as externally in collaboration with others. The main factors hindering the initiation process were limited time within the office and/or difficulties finding staff with the required competence and qualities. Finding the right means to finance the intervention was sometimes difficult, often because of joint financial responsibilities between municipalities and county councils.

#### Organizational position

The informants’ position in their organization differed, which was reflected in their approach and advocacy opportunities when initiating the dance intervention. Half of the respondents had a strategic position in the field of public health. They had the mandate, knowledge and/or the assignment to implement the intervention, and a few informants could take the decision to implement the project themselves. For informants not in a decision-making position, it was sometimes hard to convince leaders about the importance of the intervention, and several attempts had to be made to persuade management to implement the intervention.

Yes, it has gone well, it has, it takes a lot of work to keep relationships going. […] [We] found people who were interested in this who were managers or supervisors or who had authority from them so it could be carried it out in a good way. (IP8)

#### Collaborative strategies

Most of the informants expressed that establishing the idea within the organizations as well as at the political level played a vital role in the outcome of the implementation of the dance intervention. To achieve durability in the project, financial resources and devoted staff were described as key components.

… it is so important that you build up a foundation like we have done. Do the prep work, and then make sure that each stage is anchored in it. (IP8)

The informants described different collaborative arrangements between and within municipalities and county councils as necessary for the implementation of the dance intervention. For example, coordinating groups were specially set up for the implementation process. Collaboration arose exclusively where the dance project was adopted and appeared in diverse ways depending on the resources within the organization. The informants expressed a wish to collaborate across organizational boundaries but described the structure for collaboration between the organizations as vague. Some of the respondents claimed that none of the organizations wanted to take on the main responsibility or be in charge of the financial resources. They also experienced diverse opinions regarding the responsibility for the intervention and/or target group.

No, then I said that I thought it sounded interesting but that there needed to be a few others from the county council involved; it can’t just be put on the municipality I thought. (IP3)

A key element in the original dance intervention is that it was free of charge for the participating girls, including transportation to the dance classes. Because of the societal structure in Sweden, in most scenarios described by the informants, the dance intervention thus required shared financial responsibility between county councils and municipalities. The joint financial responsibility was described as a predicament by some informants.

#### Personal competence and qualities

The informants described the recruitment of a dance instructor with special professional and personal qualities as a key factor in the outcome of the dance intervention. Social skills and the ability to create a sense of undemanding kinship with the girls seemed critical. Skills such as empathy and motivation were also key features. For some of the informants, the recruitment of a suitable dance instructor proved to be a challenge.

… I am completely convinced that even if you recruit a really competent dance teacher who doesn’t have these qualities or social skills, or even some of them, you are not going to get the same results. (IP1)

#### Time constraints

Some informants described that the time required to recruit a person with the right qualities to start the dance course for instructors was limited. Others described that they had to act within a limited time-frame to get the intervention started. The time limits were described by the informants as negative for the results of the implementation.

I had about two weeks to try to recruit teachers for the course so we were really, really short of time. […] then we found out, probably through rumours, that it was the last time the course would be held. (IP4)

#### Reach of the intervention

Informants expressed difficulties regarding recruitment of girls who were eligible to take part in the dance groups. The target group was small and sensitive to dropout. To recruit participants to the dance groups, exceptions from the original intervention were made and, for example, girls without internalizing problems were included. Other informants described that they were eager to make sure that the intervention was followed as intended. Further, some informants struggled with equality issues and felt they had to offer the intervention to boys as well.

… we could relax the rules a little too; then we could say that all the girls who wanted to could join in. (IP10)

Another concern described by some informants was that if the dance intervention included only a small group of girls, the intended public health effect would not be reached. Projects aimed at a larger group of young people seemed preferable in order to decrease the prevalence of psychological health problems among adolescents.

## Discussion

The aim of this study was to explore experiences of the diffusion and initiation of a public health intervention targeting adolescent girls with internalizing problems, i.e., stress and psychosomatic symptoms. The informants mentioned several factors that may have had an impact on the diffusion of the dance intervention. There was a demand for some kind of intervention aimed at adolescent girls; the dance intervention was perceived as evidence based and compatible with the informants’ beliefs and values. Further, information regarding the intervention was packaged in an appealing format and was easy to pass on. The initiation was hindered and/or facilitated by local conditions concerning, for instance, organizational factors, recruitment of dance instructors and participants, and time constraints.

### Perceived appeal and trustworthiness

Some of the results in our study can be understood with reference to Rogers’ diffusion of innovations theory (Rogers, 2003) and subsequent implementation frameworks such as the ecological framework constructed by Durlak and DuPre (2008) and the Consolidated Framework for Advancing Implementation Research (CFIR) developed by Damschroder et al. (2009). The perceived relative advantage, i.e., the degree to which an innovation is perceived as better than what it supersedes, is one of the key elements in Rogers’ theory. In the extensive framework and review conducted by Durlak and DuPre (2008), the perceived need for an innovation as well as perceived benefits of the innovation desired at the local level were found to be consistently related to positive implementation results. Further, the informants expressed that the expected benefits of the innovation came at a relatively low cost, with the perceived promise of rapid results for the target group (Philipsson et al., 2013). This perception explains the interest in the innovation. Providers who see a specific need for an intervention, believe that the intervention can produce benefits, and have the skills and confidence to do what is expected from them have been found to be more likely to implement intervention programmes with a high level of fidelity (Durlak & DuPre, 2008).

In this study, the respondents expressed that they were particularly susceptible to the innovation at hand because it filled a gap between pressing demands from the political leadership calling for action for adolescent girls with psychological health problems and available public health innovations targeting this group. This fits well with what is labelled tension for change in the CFIR. Tension for change is part of the implementation climate; if the current situation is perceived as intolerable by stakeholders and there is a need for change, this contributes to a positive implementation climate (Damschroder et al., 2009). Further, the results in the present study show that the scope of the intervention was in concordance with individual and personal beliefs. This reflects compatibility, a sub-construct of implementation climate (Damschroder et al., 2009), which is also an important factor for diffusion (Rogers, 2003). Similar results have been reported regarding the implementation of another dance intervention where the stakeholders’ personal beliefs regarding the effects of the intervention were important for convincing the organization to implement and support the dance intervention (Demers, Thomas, Wittich, & McKinley, 2015).

The dance intervention was perceived as comprehensible and relevant by the informants. Further, it was perceived as a realistic and practically possible intervention to implement. This reflects that the intervention was feasible, i.e., that it can be used within the given context (Proctor, Silmere, Raghavan, Aarons, & Griffey, 2011). In the taxonomy by Proctor and colleagues, feasibility is distinguished from appropriateness (or compatibility), as appropriateness is the perceived fit of an innovation while feasibility is the actual fit in terms of, for example, resources or other requirements (Proctor et al., 2011).

### Convenient information

The rate of adoption was infused by the way in which information could be passed on to others. The argument that the intervention was based on research gave confidence to the advocates for the innovation and was used to convince peers and co-workers to adopt the innovation. However, the research results, or the fact that there was only one available effect study, were not questioned or critically addressed by any of the respondents, possibly indicating a high trust in scientifically demonstrated results. The study results were available as popular science, and the intervention itself as well as the results were presented in a straightforward manner. According to Damschroder et al. (2009), ease of access to digestible information regarding an intervention and how to use it practically in organizations increases the readiness for implementation. The information and knowledge can come from experts, staff, documents and computerized systems. Regarding the dance intervention, information from experts was in the form of both research literature and a film clip. The importance of design and packaging is a construct from CFIR that seems to be less well addressed than other constructs in implementation studies (Ilott, Gerrish, Booth, & Field, 2013). However, in the present study, this seemed to be of particular importance in the rapid diffusion of the intervention.

### Contextual factors

The informants mentioned several difficulties that arose during the implementation process. Overall, informants with a mandate to make decisions regarding the implementation obviously had an advantage in organizing dance groups. For others, issues with finding suitable co-workers and partners between community and municipality, as well as finding solutions regarding funding and solving practical problems, were time consuming. Lack of support from the organization has been reported as a key barrier to implementation (Demers & McKinley, 2015). Some informants reported that collaborative strategies were useful to carry through the dance intervention, which can be understood with reference to several implementation frameworks highlighting cooperation and collaboration among local agencies (Durlak & DuPre, 2008).

Fidelity to the original intervention varied; some informants described high ambitions to follow the original intervention, whereas others made modifications to solve different contextual problems. A few informants mentioned changing the inclusion criteria to gain a broader recruitment base. However, in a qualitative study evaluating the intervention, it was found that the girls participating in the dance intervention experienced stress relief during the dance class because it was a place where they did not feel critically evaluated, as they did in their daily life. Further, it was found that connecting with other girls with the same kind of problems as themselves contributed towards feeling comfortable in the dance group (Duberg et al., 2016). Changing the target group to include boys or girls without internalizing problems in order to recruit more adolescents to the groups may decrease the effectiveness of the intervention for the girls who need it most. The importance of a shared habitus and experiences in public health interventions has been emphasized in other intervention groups (Bunn, Wyke, Gray, Maclean, & Hunt, 2016; Coalter, 2013) and may be equally important for adolescent girls with internalizing behaviour. However, more research is needed to find out whether mixed groups can be as effective.

### Methodological considerations

This study has a number of shortcomings that must be addressed. The voluntary nature of participation in the study means that the stakeholders who chose to participate may differ from those who declined participation. Reasons for declining participation included changed work situation, perceived recall bias or a non-response to the invitation. Possibly, the individuals who accepted participation were more interested in the intervention or represented organizations where the implementation had been more successful, and they therefore had the intervention more at the forefront of their mind. The participants represented various organizations, positions, levels of work experience, genders and ages, which contributed to a large variation in the sample. This heterogeneity increases the possibility of viewing the implementation of the dance intervention from different angles, which can be considered as a strength of the study. Transparency was sought by describing the sampling procedure and data analyses in detail.

Telephone interviews were used for the data collection. A disadvantage associated with this method is the limited interaction, e.g., changes in body language and facial expressions are lost (Musselwhite, Cuff, McGregor, & King, 2007). However, the perceived anonymity associated with telephone interviews may reduce response bias due to social desirability because the participants are less affected by the presence of an interviewer (Musselwhite et al., 2007). In the present study, where the subject under discussion was not related to personal issues or thought to be sensitive to the informants, telephone interviews were deemed appropriate. Further, the method was cost-effective and ensured that the participants could be interviewed within a short time span. The interviews were considered to give rich material sufficient for the research question. As in any qualitative research, the results of the study are not generalizable to other populations. Instead, theoretical generalization was sought by comparing our results with those from other studies as well as theoretical frameworks.

To strengthen the rigour of the analysis of the material, the research group worked closely and thoroughly. The members of the multidisciplinary research team have different experiences and pre-understanding, which we consider valuable in the analytical process because it permitted different perspectives. The research team consists of professionals with different education and experiences within the fields of public health and/or implementation research, health economics, nursing, occupational therapy and contemporary dance. The group who performed this study was not involved in the research leading up to the intervention and is thus unbiased.

## Conclusion

The perceived need for an intervention to respond to a public health problem in combination with an intervention that was perceived as evidence based and effective, communicated in a comprehensible and convenient manner, led to rapid diffusion. However, the implementation was sometimes challenged by difficulties related to the pressure to offer an intervention immediately. When initiating the intervention, factors related to economy, possibility for collaboration and recruitment were of importance.
